# Cooperative Control and Its Engineering Applications in Power Systems

**DOI:** 10.1155/2014/795389

**Published:** 2014-12-30

**Authors:** Jinde Cao, Taiyou Yong, Guanghui Wen, Wenwu Yu, Xinghuo Yu, Abdulaziz Alofi

**Affiliations:** ^1^Research Center for Complex Systems and Network Sciences and Department of Mathematics, Southeast University, Nanjing 210096, China; ^2^Department of Mathematics, Faculty of Science, King Abdulaziz University, Jeddah 21589, Saudi Arabia; ^3^China Electric Power Research Institute, Nanjing 210003, China; ^4^School of Electrical and Computer Engineering, RMIT University, Melbourne, VIC 3001, Australia

In the past few years, distributed cooperative control of multiagent systems has received much attention from various scientific fields due to its wide engineering applications. A multiagent system typically contains numerous nodes and lots of links among these individual nodes. It is thus difficult or practically impossible to design a centralized controller to control all the nodes. Within this context, control of large, multiagent systems is achieved by designing some distributed controllers where only some local information is involved. One changeling issue in solving the cooperative control problem of multiagent systems is that neighboring agents may communicate with each other in a constrained communication environment. Distributed cooperative control of multiagent systems has definite meaning in analyzing and designing modern power systems. It has been known that recent trends in the modernization of power systems require communication networks that support the inclusion of new devices, for example, smart meters, and intelligent electronic devices, to reduce operation and maintenance costs, and integrate distributed renewable energy sources. In this case, the centralized analysis and control techniques for power systems are inapplicable.

The main aims of this special issue are to present analysis methods for cooperative control of multiagent systems and discuss their potential applications in modern power systems. Call for papers has been carefully prepared by the guest editors and posted on the journal's web page, which has received much attention from researchers in different scientific communities. We have received 15 papers in related research fields. All manuscripts submitted to this special issue went through a thorough peer-refereeing process. Based on the anonymous reviewers' reports, 7 original research articles are finally accepted.

We hope that the papers published in this special issue will be useful to researchers in the fields of distributed cooperative control and power systems. We also hope that the published papers will arouse further research in the topics presented as well as in the other related topics.

